# Proof-of-concept phase II study protocol to evaluate the efficacy and safety of prednisone in the treatment of idiosyncratic hepatotoxicity and its mechanistic pathways through an integrative analysis: the DILICORT clinical trial

**DOI:** 10.3389/fphar.2026.1963613

**Published:** 2026-10-26

**Authors:** Vanesa Garrido-Rodríguez, Ángela Remesal-Doblado, Miren García-Cortés, Hao Niu, Jose Cárdenas-Quesada, José M. Pinazo-Bandera, Juan Pedro Toro-Ortiz, Elena Pérez-López, Rocío Moreno-González, Elvira Bonilla-Toyos, Daniel E. Di Zeo-Sánchez, Gonzalo Matilla-Cabello, Marina Villanueva-Paz, Raúl J. Andrade, M. Isabel Lucena, Ismael Alvarez-Alvarez, Judith Sanabria-Cabrera, Mercedes Robles-Díaz

**Affiliations:** 1 IBIMA Plataforma Bionand, Instituto de Investigación Biomédica de Málaga, Servicios de Aparato Digestivo y Farmacología Clínica, Hospital Universitario Virgen de la Victoria, Departamentos de Medicina y Dermatología y de Farmacología y Pediatría, Universidad de Málaga, Málaga, Spain; 2 Centro de Investigación Biomédica en Red de Enfermedades Hepáticas y Digestivas (CIBERehd), Madrid, Spain; 3 Unidad de Investigación Clínica y Ensayos Clínicos, Instituto de Investigación Biomédica de Málaga y Plataforma en Nanomedicina, Málaga, Spain; 4 Plataforma de Investigación Clínica, Spanish Clinical Research Network (SCReN), Instituto de Salud Carlos III, Madrid, Spain

**Keywords:** clinical trial, corticosteroids, drug-induced liver injury (DILI), idiosyncratic hepatotoxicity, placebo-controlled, proof-of-concept

## Abstract

**Introduction:**

Idiosyncratic drug-induced liver injury (DILI) is an unpredictable adverse reaction ranging from mild liver enzyme elevations to acute liver failure, with management largely limited to withdrawal of the causative drug. An international propensity score-matched study of 724 DILI cases associated corticosteroid therapy with a higher liver enzyme normalization rate, particularly in moderate-to-severe cases without early improvement. These findings informed the design and selection of endpoints for the DILICORT trial, which aims to evaluate the efficacy and safety of prednisone in patients with DILI. The trial will also investigate the effects of treatment on liver function recovery and identify biomarkers associated with therapeutic response.

**Methods:**

DILICORT (EUCT 2024-513004-33–00; NCT06251232) is a national, randomized, double-blind, placebo-controlled phase II proof-of-concept trial. Sixty patients with moderate-to-severe DILI who show no ≥ 15% reduction in alanine aminotransferase (ALT) or have persistently increasing total bilirubin levels within 5–10 days will be recruited across 15 Spanish centers and followed for 12 weeks. Participants will be randomized to receive a 5-week course of prednisone or matching placebo. The primary objectives are to evaluate prednisone efficacy, assessed by the time to bilirubin reduction or normalization, and its safety and tolerability. Secondary objectives include changes in ALT, aspartate aminotransferase and international normalized ratio, progression to acute liver failure, and health-related quality of life. In addition, there are mechanistic objectives focused on elucidating DILI pathogenesis and biomarker discovery.

**Discussion:**

DILICORT is the first multicenter, randomized, double-blind, placebo-controlled trial of prednisone in acute idiosyncratic DILI, designed to provide the highest evidence for this therapeutic approach. It specifically targets patients with moderate-to-severe DILI who do not show a favorable early clinical course and incorporates centralized adjudication of suspected DILI cases. Its integrated mechanistic approach may improve understanding of DILI pathophysiology, identify prognostic and treatment-response biomarkers, and facilitate the development of precision medicine strategies and evidence-based clinical guidelines.

**Clinical Trial Registration:**

https://www.clinicaltrialsregister.eu/ctr-search/search, identifier EUCT 2024-513004-33-00; https://clinicaltrials.gov/, identifier NCT06251232.

## Graphical Abstract

**Figure:**
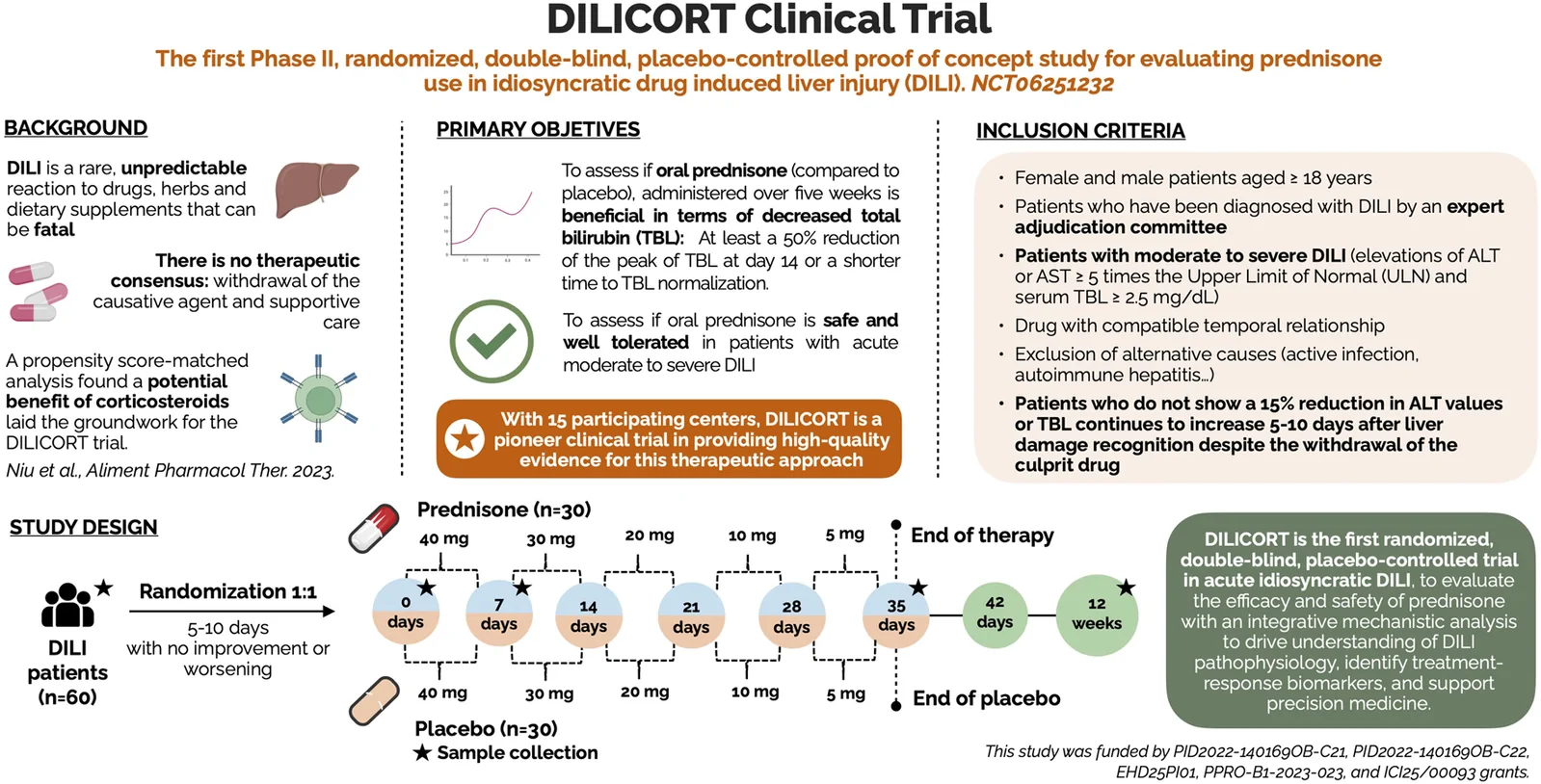

## 1 Introduction

Idiosyncratic drug-induced liver injury (DILI) is a rare and unpredictable form of hepatotoxicity following exposure to drugs typically safe for most individuals (Andrade et al., 2019a; Andrade et al., 2019b). The impact of DILI is global, as it is observed across all age groups, races, and with a wide range of therapeutic drugs, with a remarkable increase due to herbal and dietary supplements (Chalasani et al., 2015; Shen et al., 2019). The severity of DILI can range from asymptomatic hypertransaminasemia to acute liver failure and death, with most patients presenting jaundice, abdominal pain or nausea (Stephens et al., 2021). Even though DILI is associated with significant morbidity and mortality, no treatments with proven efficacy are available (Stephens et al., 2021).

The pathophysiology of idiosyncratic DILI is complex and multifactorial. Proposed mechanisms include impaired hepatic drug transport, generation of reactive metabolites, mitochondrial and endoplasmic reticulum stress, and disruption of bile acid homeostasis (Andrade et al., 2019b). These stress signals may trigger innate and adaptive immune responses that determine the progression from initial cellular injury to clinically apparent liver damage (Cueto‐Sanchez et al., 2021). The immune-mediated nature of many DILI instances is further supported by the recurrence of liver injury upon re-exposure to the offending agent due to persisting cell-mediated immunity (Mosedale and Watkins, 2017), as well as the association of DILI risk with HLA alleles encoding antigen-presenting molecules that interact with T-cell receptors and modulators of T-cell signaling (Andrade et al., 2019b).

Diagnosing DILI is challenging due to the lack of a gold standard test, relying instead on clinical suspicion, exclusion of other potential causes and consideration of multiple diagnostic elements (Andrade et al., 2019a). International collaborative efforts, including the pioneering Spanish DILI Registry, have significantly contributed to systematic case validation and recognition of disease patterns (Stephens et al., 2021).

Given the immune-mediated nature of many DILI instances, corticosteroids represent an appealing therapeutic option since they are potent immunosuppressive agents and have demonstrated efficacy in the treatment of immune-mediated liver inflammation in conditions including autoimmune hepatitis (AIH) and alcohol-related hepatitis. However, they are not devoid of systemic adverse effects. Indeed, physicians sometimes prescribe systemic steroids to treat more severe DILI cases and those associated with extrahepatic manifestations, despite the absence of large systematic studies evaluating corticosteroid use in patients with suspected idiosyncratic DILI (Andrade et al., 2019a).

Limited retrospective data suggest that the use of corticosteroids in more severe DILI cases is associated with more rapid recovery of serum bilirubin levels (Wree et al., 2011; Hu et al., 2016). Conversely, other retrospective series suggest an increased risk in patients receiving high-dose prednisone (>40 mg/day); however, these studies do not adequately account for selection bias, as corticosteroids are more likely to be administered to patients with more severe liver injury and hyperbilirubinemia at presentation (Wan et al., 2019). As a result, the worse clinical outcomes observed in corticosteroid-treated patients may reflect the underlying severity of the disease rather than a lack of treatment efficacy, indicating potential confounding by indication. Recently, in a carefully designed propensity score-matched analysis with 724 well-characterized DILI cases prospectively included in the Spanish DILI Registry and Indiana University School of Medicine, corticosteroid administration was not associated with an increased risk of developing acute liver failure (ALF), but patients receiving corticosteroids had a significantly higher normalization rate of liver enzymes than untreated patients (Niu et al., 2023). This study lays the groundwork for proposing the DILICORT clinical trial.

In this context, biomarker research is crucial for gaining a mechanistic understanding of DILI and exploring potential therapeutic approaches. International efforts have identified promising candidates such as osteopontin (OPN), cytokeratin-18 (K18 and ccK18) (Cueto‐Sánchez et al., 2023), macrophage colony-stimulating factor receptor-1 (MCSFR1) (Church et al., 2019) alongside circulating cell-free (cf) RNA molecules (Starkey et al., 2011), immune or metabolomic signatures (Barr et al., 2010; He et al., 2023) and more recently, circulating bile acids (Monte et al., 2026), which may enable earlier and non-invasive monitoring of disease progression and treatment response. Integrating these biomarkers into an interventional framework may also help clarify the biological mechanisms underlying corticosteroid response and improve patient stratification.

The absence of proven effective treatments creates a challenging clinical scenario with no clear therapeutic guidelines, often leading to the empirical use of corticosteroids without solid evidence. Although intravenous N-acetylcysteine is sometimes used off-label in some centers as adjunctive therapy for non-paracetamol DILI (Sanabria-Cabrera et al., 2022), its proven benefit is restricted to patients with early-stage ALF. Existing data supporting corticosteroid use in DILI come from retrospective, observational studies (Wree et al., 2011; Hu et al., 2016; Wan et al., 2019; Borlak et al., 2018) and a small number of clinical trials that vary widely in DILI phenotypes (Wang et al., 2022; Huang et al., 2024) (see Supplementary Material: *Search Strategy*), causative agents, corticosteroid regimens and outcome definitions, a heterogeneity that precludes a meaningful systematic review or meta-analysis. In such a scenario, a well-designed multicenter clinical trial is crucial to evaluate the effects of corticosteroids in the treatment of acute DILI, to provide the necessary evidence to inform clinical decision-making and improve outcomes. Here, we describe the DILICORT clinical trial, a phase II proof-of-concept study design to assess the efficacy and safety of prednisone in the treatment of DILI and its underlying mechanisms through an integrative analysis.

## 2 Methods and analysis

### 2.1 Main hypothesis

The administration of prednisone over 5 weeks (compared with placebo) is associated with an improvement in liver function and of biochemical parameters and is safe in patients with idiosyncratic DILI after 5 weeks of treatment.

### 2.2 Exploratory hypothesis

Serum biomarkers, cf-RNA profiles, lymphocyte populations, plasma metabolites, gut microbiota, mitochondrial dysfunction biomarkers and magnesium levels will differ during the DILI episode between patients treated with prednisone compared to those receiving placebo, as well as between responders and non-responders.

### 2.3 Objectives

The overall aim of this study is to evaluate the efficacy and safety of corticosteroids in the treatment of moderate to severe DILI.

Primary objectives include:

- To assess whether oral prednisone, compared with placebo and administered over 5 weeks, is beneficial in terms of reducing total bilirubin (TBL), defined as a reduction of at least 50% from the peak TBL level by day 14, and shortening the time to normalization of TBL levels.
- To assess if oral prednisone (compared to placebo) is safe and well tolerated in patients with acute moderate to severe DILI.

Secondary objectives include:

- To assess if oral prednisone (compared to placebo), administered over 5 weeks, is beneficial in reducing peak alanine aminotransferase (ALT), aspartate aminotransferase (AST) and International Normalized Ratio (INR) values by at least 50% at 7 days or reducing the time to normalization.
- To assess if oral prednisone (compared to placebo), decreases the proportion of patients that evolve to acute liver failure.
- To evaluate changes in health-related quality of life with oral prednisone (compared to placebo).
- To characterize the biological mechanism underlying DILI and corticosteroid response through the integration of serum and molecular biomarkers to enable non-invasive disease monitoring and improved patient stratification.

### 2.4 Study design

This is a national, multicenter, proof-of-concept phase II randomized, double-blind, placebo-controlled study (see Figure 1, EUCT: 2024–513004–33–00 and ClinicalTrials.gov: NCT06251232).

**FIGURE 1:**
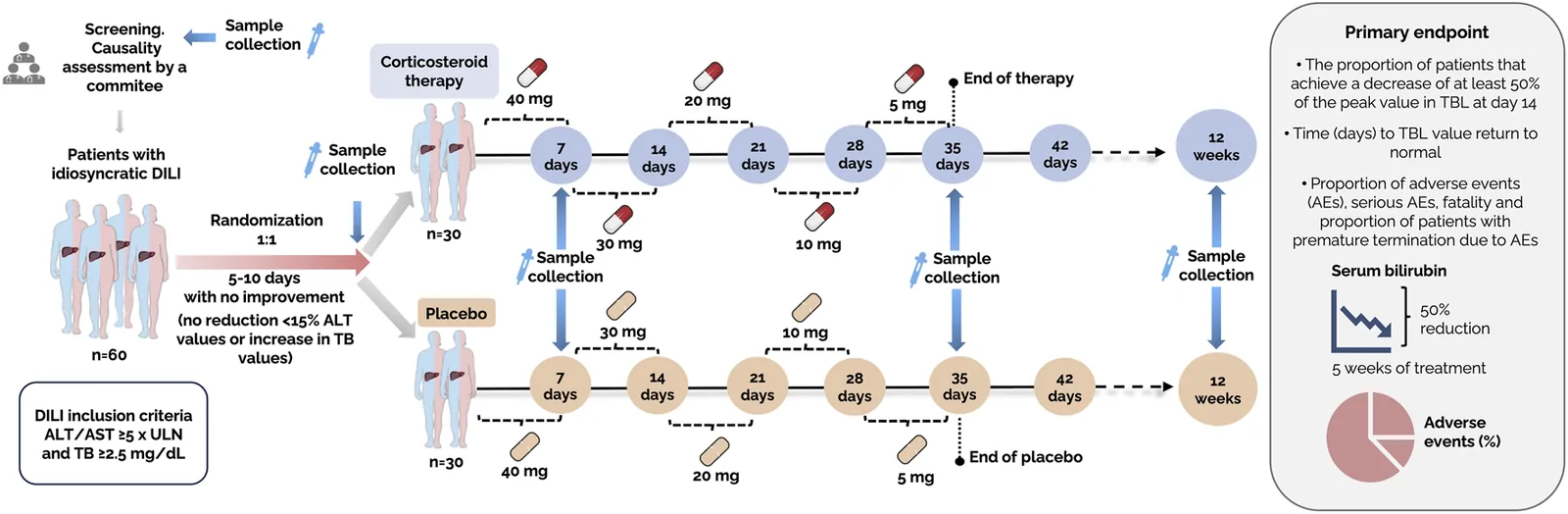
Study flow diagram outlining screening, allocation to prednisone or placebo, schedule sample collections and follow-up periods.

The recruitment period began in May 2024, and the trial, including follow-up period, is expected to be completed by December 2028 (estimated). The study duration per patient includes up to 5 weeks for treatment period, and 7 weeks for the follow-up period; thus, each patient will be followed within the study for 12 weeks from inclusion. Patients will be followed up until all liver parameters normalize (<1 fold the upper limit of normal (ULN)), or otherwise up to 1 year in the clinical setting outside of this study protocol.

### 2.5 Study population and recruitment

Sixty male and female patients aged ≥18 years with suspected acute DILI will be enrolled. Participants will be recruited by clinicians in hospital, outpatient clinics or emergency departments.

#### 2.5.1 Inclusion criteria

To be included in the study, patients must meet all the inclusion criteria described below:

- Female and male patients aged ≥18 years old.
- Patients who have been diagnosed with DILI by the expert committee.
- Patients with moderate to severe DILI (elevations of ALT or AST ≥5 times ULN and serum TBL ≥2.5 mg/dL).
- Patients who do not show a 15% reduction in ALT values or TBL continue to increase 5–10 days after liver damage recognition despite the withdrawal of the culprit drug.

#### 2.5.2 Exclusion criteria

Patients included in the study cannot meet any of the following exclusion criteria:

- No clear DILI diagnosis after an expert committee DILI assessment.
- DILI due to immune-checkpoint inhibitors.
- Presence of active infection as evidenced by positive urine or blood culture.
- Acute liver failure (INR >1.5 and hepatic encephalopathy).
- Model for End-Stage Liver Disease (MELD) ≥30.
- Known hypersensitivity to prednisone or placebo components.
- Pregnant or nursing mothers.
- Co-existing infection with hepatitis C, hepatitis B, or human immunodeficiency virus (HIV).
- Patients already receiving systemic steroids or other immunosuppressants.
- Inability to provide informed consent and absence of a personal consultee.
- Presence of clinically significant comorbid illnesses (by clinician’s criteria) that might impede completion of the study, or that could be worsened due to prednisone treatment (peptic ulcer, acute or chronic bacterial infections, systemic mycosis, difficult-to-control hypertension, diabetes mellitus, osteoporosis, psychiatric disorders, osteomalacia, and open-angle and angle-closure glaucoma).

### 2.6 Interventions

Patients diagnosed with DILI will be randomized into two parallel groups to receive either prednisone or placebo (tablet form). An authorized laboratory will supply 5 mg, 10 mg and 30 mg prednisone tablets/matching placebo. The prednisone/matching placebo will be provided in pillboxes packed with the exact number of pills per week.

Patients will start the medication (prednisone/matching placebo) at visit 1 according to the following posology:

- Week 1 of treatment: 1 tablet of 30 mg + 1 tablet of 10 mg daily
- Week 2 of treatment: 1 tablet of 30 mg daily
- Week 3 of treatment: 2 tablets of 10 mg daily
- Week 4 of treatment: 1 tablet of 10 mg daily
- Week 5 of treatment: 1 tablet of 5 mg daily

### 2.7 Outcomes

#### 2.7.1 Primary endpoint

- The proportion of patients that achieve a decrease of at least 50% of the peak value in TBL at day 14 (peak TBL was defined as the highest total bilirubin value recorded between DILI recognition and randomization (Visit 1), used as the reference value for calculating the percentage reduction in the primary and secondary endpoints); and/or,
- Time (days) to TBL value return to normal.
- Proportion of adverse events (AEs), serious adverse events (SAEs), fatality, and proportion of patients with premature termination due to AEs.

#### 2.7.2 Secondary endpoints

- The proportion of patients that achieve a decrease of at least 50% of the peak value in ALT, AST and INR at day 7.
- Time (days) for the ALT, AST and INR values return to normal.
- Proportion of patients that develop acute liver failure, need for liver transplantation or liver related death at day 28.
- Score changes in SF-36 and FRAIL quality of life validated questionnaires performed at visit 1 (day 1, start of treatment) and at visit 6 (day 35, end of treatment).
- Differences between experimental and placebo groups in the longitudinal profile of biomarkers and other mechanistic parameters across study visits.

### 2.8 Sample size calculation

The sample size calculation was performed using the hazard ratio (HR) as the measure of effect. An HR of 2.80 was assumed based on a previous observational study evaluating the effect of corticosteroids on the rate of liver parameters normalization (Niu et al., 2023). Assuming a 1:1 randomization allocation ratio (exposure probability of 0.5), an anticipated overall event rate of 0.65, an alpha level of 5% (two-sided), and a power of 80% using the Schoenfeld formula, a minimum of 30 primary events are required. Consequently, a net sample size of 47 patients is required to observe the necessary number of events. This translates to an estimated required sample size of 52 patients (26 per group) after accounting for a 10% loss to follow-up (Schoenfeld, 1981; Schoenfeld, 1983). Given the pilot and proof-of-concept nature of the study, a final target of 60 patients (30 per group) was deemed feasible and sufficient to provide valuable efficacy and mechanistic insights. A network of 15 experienced clinical centers involved in the Spanish DILI Registry makes the recruitment goal feasible, and additional centers can be added if needed. Finally, there are no prospective or randomized preliminary data to inform a predetermined sample size, so this estimation is based on realistic enrolment targets, a mechanistic approach, and the assumption of a large effect size.

### 2.9 Randomization and blinding

The randomization list will be generated with a validated program using blocks of four to allocate patients to the experimental and control arms. Eligible patients will be assigned to these 2 groups in a treatment allocation ratio of 1:1, 30 participants to the prednisone group and 30 participants to the placebo group. Study team and participants (patients, investigators, site personnel, laboratory personnel, sponsor, sponsor’s representatives and others involved) will be blinded to treatment assignments. Randomization will be carried out centrally and in a blinded fashion, using the module of randomization included in the electronic case report form (eCRF), so that after entering the participant data, a blinded treatment code is automatically provided, with screen view and confirmation email. Prior to including the first participants, a blinding plan will be in place, agreed by the sponsor/their representative and the study coordinator. As an additional measure to preserve the blinding, all personnel involved in any of the clinical assessments of the study will remain blinded during the study and up until the database has been locked/finalized. The possibility to perform a treatment unblinding in case of emergency will be available. In case of withdrawal of the patient and unblinded the treatment, the sponsor must be notified within 24 h after breaking the blind. The cause for the study discontinuation should be examined and recorded. Lifting the treatment blinding during the course of the study should be an exceptional measure with the sole aim to preserve the safety of trial participants. Unblinding of a participant’s treatment will lead to the discontinuation of the study medication. The statistical analysis team will remain blinded throughout the study regardless of individual unblinding events. Detailed instructions for unblinding and subsequent procedures will be provided in a dedicated Standard Operating Procedure (SOP).

### 2.10 Patient withdrawal

Participation in this study is entirely voluntary and patients may withdraw their consent at any time and for any reason without prejudice or consequence. Data collected up to the time of withdrawal will be included in the final analysis. A patient may also be withdrawn from the study if the attending clinician considers that continued participation is not appropriate, or that study treatment should be discontinued.

### 2.11 Study assessments and procedures

The study will be performed at 15 university hospitals in Spain. The study assessment and procedures are presented in Table 1.

**TABLE 1:** Chronogram of the clinical trial.

Procedures	Screening	Randomization	Follow-up						End of study
Visits	V0	V1	V2	V3^*^	V4^*^	V5^*^	V6	V7^*^	V8
Days ± Time window (days)	​	Day 1	Day 7 ± 3d	Day 14 ± 3d	Day 21 ± 3d	Day 28 ± 3d	Day 35 ± 3d	Day 42 ± 3d	Day 84 ± 7d
Informed consent and identification code	X	​	​	​	​	​	​	​	​
Inclusion/exclusion criteria and adjudication committee if applicable	X	​	​	​	​	​	​	​	​
Demographic and medical history	X	​	​	​	​	​	​	​	​
Pregnancy test (women of childbearing age)	​	X	​	​	​	​	​	​	​
Medical interview	X	X	X	X	X	X	X	X	X
Clinical examination	X	X	X	​	​	​	X	​	X
Laboratory parameters	X	X	X	​	​	​	X	​	X
Autoimmune profile (if not already done after onset of liver damage)	X	​	​	​	​	​	​	​	​
Serological test (if not already done after onset of liver damage)	X	​	​	​	​	​	​	​	​
Liver imaging (if not already done after onset of liver damage)	X	​	​	​	​	​	​	​	​
Randomization	​	X	​	​	​	​	​	​	​
Collection of samples for experimental analysis	X	X	X	​	​	​	X	​	X
Medication supply	​	X	​	​	​	​	​	​	​
Study medication compliance (self-reporting/SMAQ)	​	​	X	X	X	X	X	​	​
Adverse event record	​	​	X	X	X	X	X	X	X
Concomitant medications	X	X	X	X	X	X	X	X	X
Quality of life questionnaire (SF-36/FRAIL)	​	X	​	​	​	​	X	​	​

* These visits can be phone or on-site visits.

#### 2.11.1 Visit 0: Screening period

Patients with a high suspicion of DILI, presenting with acute liver injury and meeting inclusion criteria (ALT or AST ≥5 times ULN, and serum TBL ≥2.5 mg/dL) will be pre-selected. Informed consent will be obtained, followed by eligibility assessment involving demographics, medical history, clinical examination, blood tests (including liver, coagulation, autoimmune, and serological panels), imaging and sample collection for central biobank analysis. Liver parameters will be re-evaluated 5–10 days post-injury onset. Eligible patients showing no 15% ALT reduction or rising TBL will be reviewed by an Adjudication Committee of DILI experts for study inclusion.

#### 2.11.2 Adjudication committee

The Adjudication Committee will be composed of three specialist experts in DILI. The adjudication as a DILI case will be performed with the same methodology used in the Spanish DILI Registry (Stephens et al., 2021). After committee evaluation patients will be allocated in one of the following groups:

- Patients with DILI who fulfil all inclusion criteria and no exclusion criteria. The committee confirms their inclusion in the clinical trial and can be allocated in one of the study’s arms.
- Patients who fail the screening will be followed in a separate observational group, either as patients with DILI or with liver injury due to other causes. These patients will be analyzed separately and will not constitute a comparator group for the randomized trial.

#### 2.11.3 Visit 1: Day 1 (baseline)

Eligible patients will be randomized 1:1 to prednisone or placebo. Baseline data collection includes medical history, clinical examination, laboratory tests, pregnancy test (if applicable), sample collection, and SF-36/FRAIL questionnaires. The first dose of study treatment is administered, and medication is provided for the treatment period.

#### 2.11.4 Visit 2 (Day 7 ± 3)

In this visit, a medical interview and data collection of concomitant medications, clinical examination, routine laboratory parameters, medication compliance assessment, collection of samples for experimental analysis, and recording of adverse events will be performed.

#### 2.11.5 Visits 3, 4 and 5: Follow up (Days 14, 21 and 28 ± 3, respectively)

Follow up visits will involve a medical interview and concomitant medication recording, study medication compliance and SMAQ questionnaire, as well as adverse events recording. At investigator’s discretion, these visits may be phone or on-site visits.

#### 2.11.6 Visit 6: End of treatment (Day 35 ± 3)

The end-of-treatment visit includes a full clinical examination involving medical interview, concomitant medication and clinical examination, laboratory tests, SF-36/FRAIL questionnaires, study medication compliance, sample collection for experimental analysis and adverse event recording.

#### 2.11.7 Visit 7: Follow-up (Day 42 ± 3)

This visit consists of medical interview, concomitant medication and adverse events recording. At investigator’s discretion, these visits may be phone or on-site visits.

#### 2.11.8 Visit 8: End of study (Day 84 ± 7)

This visit consists of medical interview and concomitant medications recording, clinical examination, routine laboratory parameters, collection of samples for experimental analysis and adverse events recording.

### 2.12 Statistical analysis

All analysis will be conducted using R (version 4.6.1; R Core Team). Two-sided tests at the 5% significance level will be used. Descriptive statistics will be applied to demographic, clinical, laboratory, and comorbidity data. Quantitative data will include mean ± SD or median (IQR) by group, and categorical variables will be reported as counts and percentages. Differences between groups will be analyzed using Mann-Whitney U, chi-square, or Fisher’s exact tests. Time-based quantitative changes will be assessed with Friedman’s test, and categorical changes with the Cochran’s Q test.

The primary endpoint is the proportion of patients achieving at least a 50% reduction in TBL at day 14 or time to TBL normalization. The analysis of the time to TBL normalization will be conducted using the log-rank test and adjusted using a Cox proportional hazard model (Schoenfeld, 1981; Schoenfeld, 1983). To test the proportion of patients who achieved ≥50% reduction in TBL at day 14, a chi-square or Fisher’s exact tests, with adjustments for confounders via generalized linear models (GLM) will be conducted. An intent-to-treat (ITT) approach will be used for efficacy analysis. The safety population will include all randomized participants who receive at least one dose of the study treatment or placebo (Schoenfeld, 1981; Schoenfeld, 1983). Patients who do not achieve TBL normalization by the end of study (day 84) will be censored at that date; patients who withdraw, are lost to follow-up or die from a cause unrelated to DILI before the event will be censored at the date of their last available assessment. Safety outcomes include the proportion of AEs, SAEs, fatalities, and premature terminations due to AEs, tested with chi-square or Fisher’s exact tests. The null hypothesis states no difference between groups, while the alternative suggests higher rates in the prednisone group.

The statistical approach for the analysis of the mechanistic endpoints will be based on the nature of each outcome. Additionally, patients receiving prednisone will be stratified according to TBL levels at day 14 as responders (≥50% reduction in TBL by day 14) or non-responders (<50% reduction in TBL by day 14), for additional mechanistic analyses. For outcomes assessed at a single time point, differences between the two groups (treatment and placebo) will be calculated using the Mann-Whitney U test. Where supported by the available data, longitudinal outcomes will be analyzed using mixed-effects models, keeping a minimal model complexity due to the limited sample size. These models will use all available observations, and the extent and reasons for missing data will be described. Omics analyses will be considered exploratory. In this regard, missing values resulting from analytical detection limits, quality-control procedures, or other technical factors will be assessed separately and handled using platform-specific preprocessing procedures.

Gut microbiota diversity will be assessed using richness and Shannon indices for alpha diversity and Bray-Curtis distances for beta diversity. Differences in beta diversity between groups will be assessed using PERMANOVA. Microbiome profiles will be analyzed using the *vegan* and *phyloseq* R packages. Spearman’s correlation tests will explore relationships between biomarkers and clinical parameters. Finally, a multi-omics network will be created to show significant associations (FDR <0.05), using a multivariate approach with the *mixOmics* R package.

### 2.13 Data collection/data management plan

To ensure feasibility and quality, data collection for this study will include detailed reporting procedures, including analytical methods. During the project clinical and demographic data will be collected. In addition, information about lymphocyte populations from whole blood, mitochondrial function markers, metabolomic and proteomic panels, microbiome distribution from stool samples, and Mg2+ levels from serum and plasma will be generated. This information will be used to assess the safety and reliability of the use of corticosteroids in the treatment of idiosyncratic DILI.

After recruitment, patient personal data will be removed from all data and metadata and stored in a password-protected encrypted database generated in RedCap and stored in the University of Malaga server. Data will be pseudonymized; each participant will receive a code that will only be linked to the data except in a medical emergency. All data will be generated following FAIR data principles (Findable, Accessible, Interoperable and Reusable) and prioritizing the use of accepted terminologies and coding, e.g., MedDRA for adverse reactions. Raw data from omics analyses will be uploaded to Zenodo repository after anonymizing the data. During the study, the local investigator will authorize the monitor to visit the site and facilities used for the study; to check the CRF pages to make sure they are filled in correctly, and to compare them with the original data (participant’s medical file). The follow up of the study will be carried out at regular intervals, which will be agreed by the investigator and the monitor. The data manager will make plausibility checks and issue query notes to the sites for clarification of the discrepancies. The resolved queries will be verified against source data by the monitor. After cleaning of the database, it will be locked, and data will be ready for analysis.

### 2.14 Patient and public involvement

The formulation of the research objectives and endpoints was guided by the clinical expertise of hepatologists, gastroenterologists and clinical pharmacologists with long-standing experience in DILI. In addition, the leading group has established long-term collaborations with the European Liver Patients Association (ELPA) within the framework of the Cost Action 17,112 (Prospective European Drug-Induced Liver Injury Network; https://www.cost.eu/actions/CA17112/) to explore the concerns and attitudes of patients who had suffered an episode of hepatotoxicity. The conclusions of these findings have been considered in the design and development of the clinical trial and its recruitment strategies. Furthermore, participants enrolled in the study will be informed about the main results once available, and their perspectives will be considered in shaping future dissemination activities aimed at patient communities and healthcare professionals.

### 2.15 Data monitoring and auditing

The Clinical Research and Clinical Trial Unit of the Institute for Biomedical Research of Malaga (UICEC-IBIMA) will provide planning, leadership, and coordination for the performance of this clinical study. Moreover, the UICEC-IBIMA will carry out clinical management and regulation, monitoring activities and pharmacovigilance. In addition to the sponsor’s regulatory and administrative oversight, the Pharmacovigilance Working Group of the SCReN platform (ISCIII Platform for the Support of Independent Clinical Research) will oversee adverse event reception, registration, validation, and causality assessment, expedited reporting of serious and unexpected adverse reactions to Eudravigilance, and preparation of annual and final safety reports (DSUR), independently of the participating investigator sites.

To ensure data quality, the Principal Investigator or designee will implement and maintain quality assurance and control procedures according to written standard operating procedures (SOPs) to ensure that the study is conducted, and that the data is generated, documented, and notified in accordance with the protocol. Quality control procedures will be implemented from the data entry system, and data quality control checks will be performed on the database. The investigators agree to perform the study in accordance with these ethical and regulatory requirements. They will be aware that all study related documents and data may be audited or inspected in compliance with the participant’s confidentiality and privacy and that any concerns about participant’s rights cannot be used to refuse such audits or inspections.

### 2.16 Futility analysis

A futility analysis will be conducted, once 50% of the sample size has been reached, after patients complete the study. To determine whether continuing the trial would be futile due to the lack of likelihood of obtaining significant or clinically relevant results, a difference of 10% or less in the maximum reduction in total bilirubin peak levels at 14 days and in AST, ALT, and INR peak levels at 7 days compared with the control (placebo) group is established as the futility cutoff.

### 2.17 Sponsor

As this is an independent, investigator-initiated academic clinical trial, the trial sponsor is the *Fundación para la Investigación de Málaga en Biomedicina y Salud* (FIMABIS). FIMABIS holds overall authority and responsibility for the initiation, management and financing of the trial. The sponsor is responsible for ensuring that the initial protocol, which has received the required regulatory and ethics committee approval, as well as any subsequent amendments, are submitted for approval to the Spanish Medicines Agency (AEMPS) and the local ethical committee prior to implementations and retains authority to terminate the study prematurely if warranted. Operational oversight is exercised through the UICEC-IBIMA, which coordinates ethical, monitoring, regulatory affairs and pharmacovigilance activities. The sponsor holds authority over the pharmacovigilance process, including the notification of suspected unexpected serious adverse reactions (SUSARs) to competent authorities, and the preparation and submission of the annual Development Safety Update Report (DSUR). Data management and storage are conducted under the sponsor’s supervision in compliance with applicable data protection regulation and FAIR principles.

## 3 Discussion

The management of DILI currently relies on withdrawal of the suspected offending agent and supportive care. Despite the substantial clinical burden associated with moderate-to-severe DILI, no pharmacological treatments have been conclusively validated in high-quality randomized clinical trials, and DILI remains an orphan condition in terms of approved pharmacological therapies. The only exception is intravenous N-acetylcysteine, which, in addition to being the established treatment for intrinsic DILI caused by paracetamol overdose, has been shown to improve transplant-free survival in adults with non-paracetamol DILI presenting with early-stage acute liver failure (Lee et al., 2009), although this benefit has not been confirmed in pediatric populations (de Groot et al., 2026). In this context, the DILICORT trial represents a timely opportunity to advance personalized therapeutic strategies using a well-known, off-patent medication that is already used empirically in routine clinical practice. By evaluating this widely available drug within a rigorous randomized, double-blind, placebo-controlled framework, this study aims to advance DILI management from experience-based decision-making towards evidence-based and potentially personalized therapeutic approaches.

The only systematic review and meta-analysis of randomized trials addressing the prevention and management of idiosyncratic DILI and non-acetaminophen DILI-related acute liver failure identified 22 randomized clinical trials up to 2020, all but one conducted in Asian countries (Niu et al., 2021). Overall, these trials were limited by low methodological quality, low-threshold DILI diagnostic criteria, and the absence of clinically meaningful endpoints. The first major DILI trial evaluated magnesium isoglycyrrhizinate at two doses versus tiopronin, mainly in patients with mild DILI. This study highlighted two key challenges for trial design: the high rate of spontaneous resolution, supporting the need to enroll patients with moderate-to-severe injury, and the widespread use of hepatoprotective drugs in Chinese clinical practice (Wang et al., 2019), which limits the feasibility of placebo-controlled designs. Furthermore, a search in ClinicalTrials.gov from 2015 onwards identified six trials evaluating corticosteroids in DILI, all but DILICORT conducted in Asian countries. Only two, both conducted in patients with chronic DILI in China, have been published (Wang et al., 2022; Huang et al., 2024). A separate, prospective, open-label, single center RCT of prednisolone in severe DILI (TBL ≥171 μmol/L) reported an accelerated early bilirubin decline at week 1, but no significant difference in resolution rates from week 2 onward nor in 6-month survival (Song et al., 2023), highlighting the need for blinded, placebo-controlled evidence such as that provided by DILICORT. The remaining studies are mostly open-label, active-controlled trials with no adjudication committee, variable severity criteria, no formal prognostic stratification, and heterogeneous endpoints. To our knowledge, the only ongoing trial evaluating prednisone for DILI, aside from DILICORT, is a single-center, open-label study that compares glucocorticoids in combination with hepatoprotectant against a hepatoprotectant alone (NCT04553003). It differs from DILICORT due to its unblinded and active-comparator design, as well as shorter duration regimen and more severe, biopsy-based inclusion criteria.

The DILICORT trial is grounded on robust observational evidence suggesting that corticosteroids may benefit selected patients with DILI without increasing the risk of severe outcomes. A large international, multicenter propensity score-matched analysis showed no association between corticosteroid use and ALF, coupled with a higher rate of biochemical normalization, particularly in patients with more severe DILI and delayed spontaneous recovery (Niu et al., 2023). These findings provide a solid scientific rationale for the present trial and underscore the need for confirmation in a randomized controlled setting.

The inclusion of patients with moderate-to-severe DILI represents a deliberate and clinically justified design choice. Mild DILI cases frequently resolve spontaneously after drug withdrawal, making it difficult to detect a treatment effect and potentially exposing patients to unnecessary risks (Andrade et al., 2019a). A 5–10 days observation window prior to normalization was implemented to differentiate patients experiencing spontaneous biochemical improvement and requiring no pharmacological intervention from those with persistent or worsening injury, the latter being the population most likely to benefit from treatment (Niu et al., 2023). Additionally, given the absence of a gold-standard diagnostic tests, this interval allows sufficient time for causality assessment and adjudication committee review, with exclusion of alternative etiologies, which helps reduce the risk of misclassification. Conversely, as prior observational evidence has shown, there is no association between corticosteroid use and the risk of ALF, supporting the focus on earlier, potentially modifiable stages of liver injury (Niu et al., 2023). By enrolling patients who fail to show early improvement after withdrawal of the suspected drug, DILICORT focuses on a population with unmet therapeutic needs and a higher risk of adverse outcomes, thereby maximizing the potential clinical relevance of the findings.

Given the absence of a gold-standard diagnostic test for DILI and the reliance on clinical judgement, causality assessment and exclusion of alternative etiologies, misclassification represents a major threat to the validity of DILI trials, particularly in a multicenter setting where diagnostic practices may vary across sites. To mitigate this risk, DILICORT incorporates an independent, central adjudication committee that reviews all candidate cases using standardized causality assessment criteria, blinded to treatment allocation, ensuring an accurate and homogeneous enrolment of *bona fide* DILI cases and consistent severity stratification across participating centers. This represents a methodological advance over previous DILI trials, most of which lacked formal adjudication processes and relied on heterogeneous, site-specific diagnostic criteria, increasing the risk of including misdiagnosed or non-idiosyncratic cases and limiting the interpretability of their results.

The use of placebo as the comparator is a critical methodological strength of the study. In the absence of any approved or evidence-based pharmacological treatment for idiosyncratic DILI, placebo-controlled design remains the most appropriate approach to estimate the net pharmacological effect of an intervention separating it from the natural history of the disease and providing assay sensitivity. Empirical use of corticoids in DILI is highly variable and subject to confounding by indication, particularly as more severe patients are more likely to receive treatment (Niu et al., 2023). Blinding and placebo control are therefore essential to reduce bias and ensure the validity of efficacy and safety assessments, while all patients continue to receive optimal standard supportive care.

The selection of prednisone dosing and treatment duration is based on a careful balance between clinical experience, available observational evidence and safety considerations. A daily starting dose of 40 mg in the treatment arm was chosen. This reflects clinical experience in the treatment of DILI and in established steroid-responsive inflammatory liver diseases such as AIH and alcohol-related hepatitis (European Association for the Study of the Liver, 2015; Thursz et al., 2018), as well as retrospective studies on corticosteroids therapy suggesting potential benefit in DILI. The 5-week treatment course, including a 4-week taper (weeks 2–5) following the initial week at full dose, is intended to provide sustained immunosuppression during the critical phase of liver injury while minimizing the risk of corticosteroid-related adverse effects. This tapering strategy aligns with standard hepatology practice and provides sustained immunosuppression for a period sufficient to prevent further progression of the liver injury and accelerate the recovery. In line with this rationale, safety is evaluated alongside efficacy as a primary objective with close clinical and laboratory monitoring. The duration of follow-up and assessment up to 12 weeks after randomization was deemed to be sufficient to capture the dynamics of recovery and to evaluate the benefit–risk profile of prednisone in DILI, acknowledging that longer-term outcomes or late relapses fall outside the timeframe of this protocol (Stephens et al., 2021).

The DILICORT trial is designed as a multicenter study involving 15 hospitals in Spain, thereby enhancing both internal and external validity, mitigating the inherent heterogeneity of DILI, and facilitating patient recruitment. Should recruitment challenges arise, the study design allows for the incorporation of additional participating centers to ensure adequate statistical power. The results of this study should therefore be interpreted as preliminary but essential evidence that, if supportive of efficacy and safety, will justify larger confirmatory trials and inform future guideline development.

A distinctive and innovative feature of the DILICORT trial is its extensive mechanistic component, which goes beyond conventional clinical endpoints. Quantification of serum proteins (OPN, K18, ccK18 and MCSFR), analysis of serum cf-RNA, whole-blood immunophenotyping, gut microbiota characterization, serum metabolomics, biomarkers of mitochondrial dysfunction, serum magnesium levels, and genetic variant analysis offer a unique opportunity to elucidate the pathophysiology of DILI and the biological mechanisms underlying treatment response. Notably, bile acids have emerged as promising candidate biomarkers in previous studies (Monte et al., 2026), and DILICORT provides a prospective, randomized setting to validate their clinical utility for disease monitoring and severity assessment, rather than being limited to the identification of novel candidates. These exploratory, hypothesis-generating data may help identify predictive and pharmacodynamic biomarkers and inform the future development of prognostic and treatment-selection tools to individualize therapy, monitor early response and optimize benefit–risk at the patient level, supporting future precision-medicine approaches in hepatotoxicity that will require validation in larger cohorts.

Corticosteroids also appear to modulate several of the mechanistic pathways involved in DILI. Transcriptomic and flow cytometry studies show immune alterations, such as upregulation of genes involved in lymphoid lineage, cell division and respiratory electron transport chain in non-responders to steroid treatment (Sharma et al., 2021). Indeed, glucocorticoids exert hepatoprotective effects by modulating mitochondrial pathways and enhancing mitochondrial biogenesis (Psarra and Sekeris, 2011), among other mechanisms. In addition, corticosteroids have also been proven to be capable of altering microbial composition (Tena-Garitaonaindia et al., 2022).

To our knowledge, DILICORT is the first randomized, double-blind, placebo-controlled clinical trial specifically designed to evaluate corticosteroid therapy in acute idiosyncratic DILI. If prednisone demonstrates an acceptable benefit-risk profile, this study could shift corticosteroid use in DILI from empirical practice to an evidence-based intervention, promoting standardization of care and informing future clinical guidelines. Moreover, the mechanistic insights generated may support precision-medicine strategies in hepatotoxicity. In summary, this trial addresses an unmet clinical need while laying the foundations to improve diagnosis, prognosis and individualized therapy in DILI through the rigorous integration of clinical and multi-omics data.

## 4 Ethics and dissemination

The study (protocol version 4.2, 12 September 2025) was registered under EUCT number 2024–513004–33–00 and on ClinicalTrials.gov under identifier NCT06251232 (https://clinicaltrials.gov/study/NCT06251232; initial registration date 1 February 2024). The study will be conducted according to the Declaration of Helsinki (75th WMA General Assembly, Helsinki, Finland, October 2024) and the ICH Guideline for Good Clinical Practice ICH E6 (R3), (EMA/CHMP/ICH/135/1995). All patients will provide written informed consent or that of a personal consultee in specific circumstances where the patient lacks capacity to give informed consent. This study will be conducted in accordance with Regulation (EU) *No 536/2014* and under the Spanish *Real Decreto 1090/2015 de 4 de diciembre 2015, por el que se regulan los ensayos clínicos con medicamentos, los Comités de Ética de la Investigación con medicamentos y el Registro Español de Estudios Clínicos*. Furthermore, this project complies with Spanish national legislation, including Law 14/2007 of July 3 on Biomedical Research and Royal Decree 1716/2011 of November 18, which regulate the operation of biobanks at the national level”. This project requires the use of human samples, which will be managed through the Biobank of the Public Health System of Andalusia.

Personal data will be processed in accordance with the provisions of the Regulation (EU) 2016/679 of the European Parliament and of the Council of 27 April 2016 on the protection of natural persons with regard to the processing of personal data (General Data Protection Regulation, GDPR) and the *Spanish Organic Law 3/2018 of 5 December on the Protection of Personal Data and Guarantee of Digital Rights* (LOPDGDD), which supplements and implements the GDPR.

The results of this study will be disseminated through publication in high-impact, peer-reviewed scientific journals and presentations at national and international scientific meetings in the fields of hepatology, clinical pharmacology and drug safety. Summary results will be reported to the competent regulatory authorities and made publicly available in the relevant clinical trial registries in accordance with applicable European and Spanish regulations. If appropriate, de-identified study data underlying the published results, including relevant clinical and mechanistic datasets, may be made available in appropriate scientific repositories in accordance with FAIR data principles, applicable regulations, and participant consent. All dissemination activities will be conducted ensuring scientific rigor, transparency and full protection of participant confidentiality.
