# Supplementary material for: Proof-of-concept phase II study protocol to evaluate the efficacy and safety of prednisone in the treatment of idiosyncratic hepatotoxicity and its mechanistic pathways through an integrative analysis: the DILICORT clinical trial
Source: Front Pharmacol. 2026 Oct 26;17:1963613. doi: 10.3389/fphar.2026.1963613 (PMC13761323; doi:10.3389/fphar.2026.1963613)
Supplement: Supplementary file 1 [file Supplementaryfile1.docx]

Supplementary Material

# 1 Supplementary Methods

## 1.1 Search Strategy

A systematic search was performed in ClinicalTrials.gov to identify clinical trials evaluating corticosteroid treatment in drug-induced liver injury (DILI), restricted to studies registered from 2015 onward. Two search strategies were combined: studies classified under the condition terms "Drug-Induced Liver Injury" OR "Drug-Induced Liver Damage", and studies classified under "Hepatotoxicity". After removing duplicates, records were screened sequentially to exclude studies in which DILI was not the main study condition, non-interventional designs (prospective registries, observational, and retrospective studies), and trials not involving corticosteroid treatment. Ongoing trials or those without published results were also excluded. The study selection process is summarized in **Figure S1**.

# 2 Supplementary Figures

**Supplementary Figure 1.** Flow diagram of the study selection process for clinical trials evaluating corticosteroid treatment in drug-induced liver injury, identified through ClinicalTrials.gov
